# Aquatic Chlamydiae: A Review of Their Roles in Fish Health

**DOI:** 10.3390/microorganisms13092166

**Published:** 2025-09-17

**Authors:** Basma Mahmoud-Elkamouny, Carole Kebbi-Beghdadi, Gilbert Greub

**Affiliations:** Institute of Microbiology, Lausanne University Hospital, University of Lausanne, 1011 Lausanne, Switzerland

**Keywords:** epitheliocystis, *Chlamydiales*, *Chlamydiota*, *Chlamydia*-related bacteria, *Chlamydia*-like bacteria, gill disease, *Chlamydia*, fish disease

## Abstract

Aquaculture plays a vital role in meeting the global demand for high-quality protein. However, the fish industry is challenged by infectious diseases, including gill conditions such as epitheliocystis. Epitheliocystis is characterized by cyst-like epithelial lesions, which occur in the gills of fish, and is associated with intracellular bacteria including *Chlamydia*-related bacteria. Although epitheliocystis was initially regarded as of low significance, attention is increasing due to its impact on commercially important fish species in intense farming conditions. This review evaluates the roles of aquatic chlamydiae as pathogens contributing to fish morbidity and mortality, and as members of fish microbiota. Additionally, *Chlamydia*-related bacteria are thought to be involved in complex gill disease (CGD), characterized by lamellar fusion, epithelial hyperplasia, and inflammation. Recent discoveries have expanded the diversity of *Chlamydiota* isolated from fish, with novel species such as *Candidatus (Ca.)* Panilichlamydia rohitae, *Ca.* Piscichlamydia trichopodus, and *Chlamydia vaughanii* identified in different fish hosts. Most causative agents of epitheliocystis have not yet been cultured in vitro, although *C. vaughanii*, the first *Chlamydiaceae* member isolated from fish, was successfully cultured. As *C. vaughanii* was recently shown to be able to propagate in mammalian cells, it raises concerns about its zoonotic potential, although a pathogenic role has yet to be described.

## 1. Introduction

### 1.1. Importance of Fish Farms and Financial Risks Associated with Fish Diseases

Considering the continuous increase in the human population worldwide, aquaculture is an important sector to meet the second goal described by the United Nations Sustainable Development Initiative, i.e., to achieve zero hunger. In 2020, the production of aquatic animals reached 87.5 million tons, accounting for 56% of the total global amount of animal proteins produced for human consumption [1]. However, this industry is challenged by diseases which represent a significant financial risk with predicted losses of $84 million in the US annually [2]. Moreover, in the UK, diseases in fish are estimated to represent a risk of $62 million to $1755 million USD, representing about 5.8 to 16.5% of the total value of aquaculture production in this country [3].

### 1.2. Gill Functions and Gill Diseases

The gills perform multiple important functions, including gas exchange, osmoregulation, nitrogen waste excretion, pH regulation, and hormone production [4]. Since fish and their gills are directly exposed to the aquatic environment, they are constantly at risk of being infected by bacterial and viral pathogens, infested by parasites or fungi, and/or damaged by toxins, and as gills are physically delicate and permeable (to ensure gas and electrolytes exchange), they are also vulnerable to physical injury [5]. Thus, gill diseases are the most common diseases occurring in fish. Moreover, gill diseases are severely affecting fish production in aquaculture, since gill injury or infection can prove fatal.

### 1.3. Etiologies of Gill Diseases

Gill diseases (GDs) are caused by a variety of pathologies such as cysts, cell hypertrophy, inflammation, infiltration with inflammatory cells, and lamellar fusion. Thus, there are seven distinguishable etiology-based subtypes which refer to the causative agent of infection or mechanism of injury: (1) parasitic gill disease, (2) amoebic gill disease (AGD), (3) bacterial gill disease, (4) viral gill disease, (5) zooplankton (cnidarian nematocyst)-associated gill disease, (6) harmful algal gill disease and (7) chemical/toxin-associated gill disease. Infections by viruses or bacteria and infestations by fungi or parasites remain the most common etiologies of gill diseases observed in aquaculture, since chemical and toxin-associated gill diseases may be more easily prevented.

These infections and infestations can be influenced by several environmental variables or events such as high temperature, proximity to the infection site, or high stocking density [6]. This is important since (i) with current global warming, the mean temperature of the sea and ocean is increasing, even in Nordic countries such as Norway, Iceland, Finland, and Sweden, which are highly active in salmon farming and since (ii) economic pressure on productivity leads to increase the density of fish in salmon farms.

### 1.4. Epitheliocystis Disease

One type of common gill diseases affecting fish is called epitheliocystis. It was first described in the common carp, *Cyprinus carpio*, as mucophilosis [7]. Later, the disease was reported in other fish species, and the name mucinophilus was replaced by the name epitheliocystis, derived from the cyst-like epithelial lesions, which can be observed by histopathological examination of the gills [8].

Epitheliocystis has been described in at least 90 species of fish, including both freshwater and marine species from the wild or farmed populations. Among them, species such as Atlantic Salmon and carp are of economic importance.

Epitheliocystis disease may be due to various bacteria including gamma-proteobacteria (*Ca. Endozoicomonas* spp.), beta-proteobacteria (*Ca. Ichtyocystis* spp., *Ca. Branchiomonas* spp.), and various members of the *Chlamydiota* order (see below) [9].

Although epitheliocystis is often a benign disease, it can cause inflammation of the infected tissues and epithelial hyperplasia, leading to a significant decrease in fish growth [10]. In advanced cases, known as hyperinfections, the cyst-like branchial lamellar lesions may lead to an increase in lamellar fusion followed by respiratory distress and eventually, death [11]. Predisposing factors include the presence of nutrients, high population density, season, temperature, and age of fish [9].

Many epitheliocystis-attributed losses in aquaculture take place in either the larval or juvenile stage [9,10,11,12,13]. This disease is usually regarded as of little or no significance since it only affects a limited number of species cultured for commercial purposes. However, aquaculture species are more at risk of being infected due to the higher stocking densities and greater stresses placed upon the fish [9,14]. Additionally, there are reports in Atlantic Salmon farms in Norway, where epitheliocystis is correlated with high fish mortality and potential important economic losses [15,16].

### 1.5. The Chlamydiota Order

Members of the *Chlamydiota* order are Gram-negative obligate intracellular bacteria. They are distributed worldwide and cause various diseases in humans, domestic animals, wildlife, livestock, and exotic species. The replication cycle of *Chlamydiota* occurs in two stages [17,18]. Bacteria alternate between (i) elementary bodies (EBs), which are the infectious particles that can enter eukaryotic cells by endocytosis and then reside in a cytoplasmic vacuole called “inclusion”, and (ii) reticulate bodies (RBs), that replicate by binary fission when located in the chlamydial replicative vacuole [18,19].

The taxonomic organization of the *Chlamydiota* order has changed dramatically over the last twenty years [20] and multiple new family-level lineages were described in addition to the well-known *Chlamydiaceae* family that encompasses established human and animal pathogens such as *Chlamydia pneumoniae*, *Chlamydia trachomatis*, *Chlamydia psittaci*, *Chlamydia pecorum*, and *Chlamydia abortus*.

Bacteria belonging to the new families are referred to as ‘*Chlamydia*-related bacteria’, *Chlamydia*-like organisms, or environmental *Chlamydiae*. This latter denomination is related to the large number of species isolated from an environmental source, such as water [21,22], free-living amoebae [18,23,24], or arthropods [25,26,27,28,29]. *Chlamydia*-related bacteria can also cause diseases and mortality in a wide range of species, including humans, ruminants, koalas, bats, birds, and arthropods. In fish, as said above, they cause epitheliocystis [9].

## 2. *Chlamydia*-Related Bacteria as Causative Agents of Epitheliocystis

### 2.1. Chlamydia-Related Bacteria Associated with Epitheliocystis

**Figure 1:**
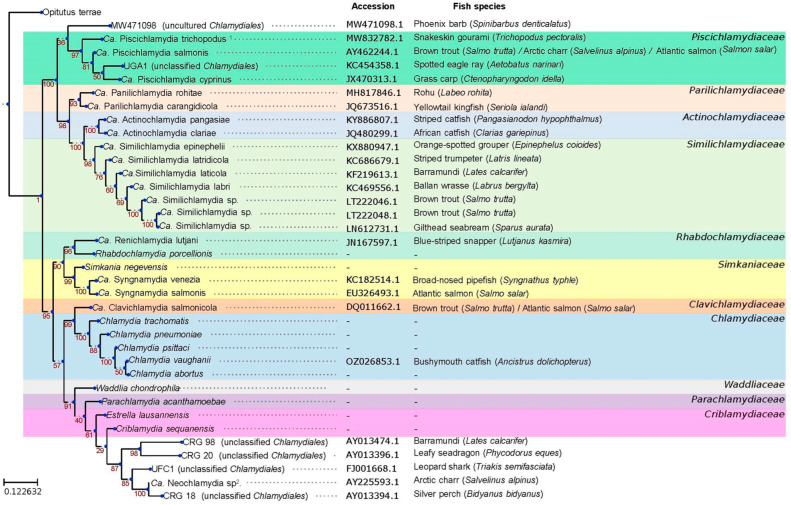
Phylogenetic tree of *Chlamydiota* species based on 16S rRNA. The sequence accession number and the host fish species are reported for fish-infecting species and background colors indicate know family-level lineages. The tree was reconstructed by neighbor-joining based on distances calculated with the Jukes-Cantor substitution model and 100 boostrap. ^1^ *Ca.* Piscichlamydia trichopodus is clearly a member of a new genus (and was thus renamed *Ca.* Trichochlamydia trichopodus) and it might even be a member of a new family-level lineage. ^2^ Considering the low similarity of its 16S rRNA sequence with known *Neochlamydia* spp., this species should be renamed “unclassified *Chlamydiales*”.

Members of the *Chlamydiota* order, which cause epitheliocystis, were described based on the sequencing of their 16S rRNA gene and were classified in eight different family-level lineages: *Parachlamydiaceae*, *Simkaniaceae*, *Rhabdochlamydiaceae*, *Candidatus* (*Ca*.) Similichlamydiaceae, *Ca*. Clavichlamydiaceae, *Ca*. Piscichlamydiaceae, *Ca*. Parilichlamydiaceae, and *Ca.* Actinochlamydiaceae. In addition, a few species are still unclassified (see Table 1 and Figure 1) [30].

Members of the *Parachlamydiaceae*, *Simkaniaceae*, and *Rhabdochlamydiaceae* families include suspected agents of epitheliocystis but can also infect free-living amoebae, mammals, insects, and arthropods. *Ca*. Similichlamydiaceae, *Ca*. Clavichlamydiaceae, *Ca*. Piscichlamydiaceae, *Ca*. Parilichlamydiaceae, and *Ca.* Actinochlamydiaceae families exclusively contain agents of epitheliocystis [20].

### 2.2. Recently Discovered Chlamydia-Related Bacteria Associated with Fish

Within the past seven years following the publication of the review by Blandford et al. evaluating causative agents of epitheliocystis [30], 4 novel *Chlamydiota* bacteria which are able to infect fish have been described. These bacteria were shown to be present in four species of fish and one of them belongs to a new family-level lineage.

***Ca*. Panilichlamydia rohitae**

In 2019, the DNA of a new species belonging to the family *Ca.* Parilichlamydiaceae was isolated in India, from the gills of a freshwater species, the rohu (*Labeo rohita*), affected by epitheliocystis. It was named *Ca.* Panilichlamydia rohitae and its identification expanded both the number of *Chlamydia*-related bacteria causing this disease and the number of host fish species affected [31].

***Ca*. Piscichlamydia trichopodus**

Then in 2022, in Thailand a new *Chlamydia*-related organism named by the authors *Ca.* Piscichlamydia trichopodus, caused a systemic intracellular infection in snakeskin gourami (*Trichopodus pectoralis*) along with a Myxozoa parasite [32]. Analysis of the 16S rRNA gene revealed 93.5% sequence similarity to a partial 16S rRNA gene from a non-cultured bacterium obtained from a Mediterranean Sea bream with gill disease, indicating affiliation to the same family-level lineage (an unclassified family) [33]. The second highest sequence similarity (92.3%) was with *Ca.* Piscichlamydia sp. associated with epitheliocystis infection in cyprinids. According to the taxonomic criteria established in 2015 by Pillonel et al., 16S rRNA similarity below 92.5% indicates two species belonging to different families [33]. Thus, *Ca.* Piscichlamydia trichopodus was wrongly affiliated into the *Ca.* Piscichlamydia genus and should instead be in a new genus-level lineage, since it exhibits only 92.3% 16S rRNA sequence similarity with *Ca* Piscichlamydia salmonis, the type strain of the *Ca* Piscichlamydia genus. However, since the 16S rRNA sequence available is relatively short and no additional gene is available, it is not possible to determine whether *Ca.* Piscichlamydia trichopodus belongs to a new family-level lineage or to a new genus but within the *Ca.* Piscichlamydiaceae family. For clarity, we decided to keep the new genus within the *Ca.* Piscichlamydiaceae family, but we propose here to rename *Ca.* Piscichlamydia trichopodous, *Ca.* Trichochlamydia trichopodus. 

Interestingly, *Ca.* Piscichlamydia trichopodus, unlike other *Chlamydia*-related bacteria, was found in the connective tissue of the fish, instead of in the epithelial cells. The presence of these bacteria near damaged cartilaginous tissue suggests that this new pathogen requires cartilage for its metabolism [32].

**Uncultured member of the Chlamydiota order in cyprinids**

In 2022, a new *Chlamydia*-related species belonging to a new family was isolated in China from cultured cyprinids (*Spinibarbus denticulatus*). This was the first case reported of epitheliocystis in cultured fish in China and it caused 40% mortality [34].

***Chlamydia vaughanii***

Finally, a novel species belonging to the *Chlamydiaceae* family, *Chlamydia vaughanii*, was isolated in our lab in 2022, from a dead tropical fish, *Ancistrus dolichopterus* [35]. Although bacteria of the *Chlamydia* genus were previously isolated from birds, reptiles and mammals, *C. vaughanii* was the first member of this genus isolated from a fish. Bacterial infection was the probable cause of fish death, but histological examination of gills was not conclusive for the presence of cysts thus preventing formal identification of epitheliocystis. *C. vaughanii* has a larger genome (1.3 Mb) than other members of the *Chlamydia* genus, which is due to multiple duplications in genes encoding putative adhesins.

We demonstrated that *C. vaughanii* has the capacity to propagate in mouse cells and in a fish cell line (EPC175) grown at 30 °C but not in insect cells nor in amoebae [35]. Moreover, recently obtained data, yet unpublished, indicate that this bacterial species can multiply in several human cell lines, including macrophages. This capacity of spanning the species barrier is worrisome regarding human health and may be due to its expanded adhesin gene reservoir, enlarging the bacterial tropism [35].

To date, it has not been possible to culture any of the epitheliocystis agents identified in fish, thereby hampering the reproduction of the disease in an experimental model [9,30,36,37,38]. *Chlamydia vaughanii* is, thus far, the only *Chlamydiota* bacteria isolated from fish that was successfully retrieved in cell culture and whose host range can be investigated in vitro (see below).

**Table 1:** *Chlamydiota* bacteria in fish.

Fish Species	Family-Level Lineage	*Chlamydiota* Species	Accession	References
African catfish (*Clarias gariepinus*) *	*Actinochlamydiaceae*	*Ca.* Actinochlamydia clariae	JQ480299	[39]
Arctic charr (*Salvelinus alpinus)* *	*Piscichlamydiaceae*	*Ca.* Piscichlamydia salmonis	AY462244	[40]
	*Parachlamydiaceae*	*Ca.* Neochlamydia sp.	100% identical to AY225593.1.	[10]
Atlantic Salmon (*Salmo salar*) **	*Clavichlamydiaceae*	*Ca.* Clavichlamydia salmonicola	DQ011662	[41,42,43]
	*Simkaniaceae*	*Ca.* Syngnamydia salmonis	EU326493	[44]
	*Piscichlamydiaceae*	*Ca.* Piscichlamydia salmonis	AY462244	[15,43,45,46]
Ballan wrasse (*Labrus bergylta*) **	*Parilichlamydiaceae*	*Ca.* Similichlamydia labri	KC469556	[47]
Barramundi (*Lates calcarifer*) **	*Parilichlamydiaceae*	*Ca.* Similichlamydia laticola	KF219613	[48]
	not determined	CRG 98 (unclassified *Chlamydiales*)	AY013474	[37]
Blue-striped snapper (*Lutjanus kasmira)* **	*Rhabdochlamydiaceae*	*Ca.* Renichlamydia lutjani	JN167597	[49]
Broad-nosed pipefish (*Syngnathus typhle*) **	*Simkaniaceae*	*Ca.* Syngnamydia venezia	KC182514	[50]
Brown trout *(Salmo trutta)* *	*Piscichlamydiaceae*	*Ca.* Piscichlamydia salmonis	AY462244	[43,51]
	*Clavichlamydiaceae*	*Ca.* Clavichlamydia salmonicola	DQ011662	[41,42,43,46,51,52]
	*Parilichlamydiaceae*	*Ca.* Similichlamydia sp.	LT222046 LT222048	[51]
Bushymouth catfish *(Ancistrus dolichopterus*) *	*Chlamydiaceae*	*Chlamydia vaughanii*	OZ026853.1	[35]
Common carp (*Cyprinus carpio*) *	*Parachlamydiaceae*	*Ca.* Neochlamydia sp.	not available	[53]
	*Parachlamydiaceae*	*Ca.* Protochlamydia sp.	not available	[53]
	*Piscichlamydiaceae*	*Ca.* Piscichlamydia sp.	not available	[53]
Gibel carp (*Carassius auratus*) *	*Parachlamydiaceae*	*Ca.* Neochlamydia sp.	not available	[53]
	*Parachlamydiaceae*	*Ca.* Protochlamydia sp.	not available	[53]
	*Piscichlamydiaceae*	*Ca.* Piscichlamydia sp.	not available	[53]
Gilthead seabream (*Sparus aurata*) **	*Parilichlamydiaceae*	*Ca.* Similichlamydia sp.	LN612731	[54]
Grass carp (*Ctenopharyngodon idella)* *	*Piscichlamydiaceae*	*Ca.* Piscichlamydia cyprinus	JX470313	[55]
Leafy seadragon *(Phycodorus eques)* **	not determined	CRG 20 (unclassified *Chlamydiales*)	AY013396	[37]
Leopard shark *(Triakis semifasciata)* **	not determined	UFC1 (unclassified *Chlamydiales*)	FJ001668	[56]
Orange-spotted grouper (*Epinephelus coioides*) **	*Parilichlamydiaceae*	*Ca.* Similichlamydia epinephelii	KX880947	[57]
Phoenix barb (*Spinibarbus denticalatus*) *	not determined	MW471098 (uncultured *Chlamydiales*)	MW471098	[34]
Rohu (*Labeo rohita*) *	*Parilichlamydiaceae*	*Ca.* Panilichlamydia rohitae	MH817846	[31]
Silver perch (*Bidyanus bidyanus*) *	not determined	CRG 18 (unclassified *Chlamydiales*)	AY013394	[37]
Snakeskin gourami (*Trichopodus pectoralis)* *	new family-level lineage	*Ca.* Piscichlamydia trichopodus ^1^	MW832782	[32]
Spotted eagle ray (*Aetobatus narinari*) **	not determined	UGA1 (unclassified *Chlamydiales*)	KC454358	[58]
Striped catfish (*Pangasianodon hypophthalmus*) *	*Actinochlamydiaceae*	*Ca.* Actinochlamydia pangasiae	KY886807.1	[59]
Striped trumpeter (*Latris lineata*) **	*Parilichlamydiaceae*	*Ca.* Similichlamydia latridicola	KC686679	[60]
Yellowtail kingfish (*Seriola ialandi)* **	*Parilichlamydiaceae*	*Ca.* Parilichlamydia carangidicola	JQ673516	[61]

* Fresh water fish, ** Marine water fish. ^1^ Corresponds to a new genus-level lineage and was thus renamed *Ca.* Trichochlamydia trichopodus.

## 3. *Chlamydia*-Related Bacteria May Play a Role in Multifactorial Complex Gill Disease

Recently, there has been an increase in the number of cases of multifactorial or non-specific diseases called ‘Complex Gill Disease’ (CGD). CGD commonly affects farmed fish and arises from concurrent infections by multiple putative pathogens with mixed etiologies [62]. There are two types of CGDs, proliferative gill disease (PGD) and proliferative gill inflammation (PGI) [5].

### 3.1. Proliferative Gill Disease

PGD is a non-specific term originated from the examination of gross lesions in the gills of salmon. Additionally, PGD has been used to refer to histological proliferation (i.e., lamellar epithelial cell hyperplasia and fusion of adjacent lamellae), but the term does not describe a specific syndrome or disease [5]. *Ca.* Piscichlamydia salmonis was reported to be associated with PGD in Norway [63]. This study evaluated three fish farms, two marine and one freshwater. The two marine farms experienced about 80% losses due to PGD, where *Ca*. Piscichlamydia salmonis, *Neoparamoeba* sp., and Salmonid gill pox virus (SGPV) were identified in the gills of the fish. The freshwater farm had about 20% losses attributed to PGD, but SGPV was the only pathogen detected. Thus, the pathogenic role of the Pox virus is obvious. It is likely that *Ca*. Piscichlamydia salmonis was also involved in the observed PGD in marine salmon but given the concomitant presence of a protist and the Pox virus, the 2nd Koch’s postulate is not fulfilled, and it is not possible to precisely define the respective role played by each protagonist in the pathogenesis of the observed gill lesions.

### 3.2. Proliferative Gill Inflammation

PGI was the term used to report recurrent GD outbreaks which took place in salmon farms on the southwest coast of Norway. These outbreaks mostly affected smolts (young salmon), which had been transferred to the sea the previous spring [6]. The young Atlantic Salmon were transferred into 12 seawater farms located in the mid- and southwest of Norway and sampled for a year. PGI outbreaks were evaluated by clinical examination, mortality data, and histology and were diagnosed in six of seven farms in southwest Norway. There was no PGI recorded in the five farms located in mid-Norway. Mortality started three to five months after the transfer to seawater and outbreaks lasted about one to three months. *Ca*. Piscichlamydia salmonis was detected by real-time PCR exclusively in fish from farms affected by PGI, with findings indicating an association between *Ca.* Piscichlamydia salmonis bacterial load and the severity of PGI. The histological changes which occurred were epithelial cell proliferation, inflammation, necrosis, and vascular changes such as lamellar hemorrhage and/or lamellar thrombosis. Many other microbial causative agents have been associated with PGI [5].

### 3.3. Proliferative Gill Inflammation and Epitheliocystis

PGI may evolve into a classical epitheliocystis disease (as described above, with the presence of cysts in the gills). Conversely, a chronic epitheliocystis disease may evolve into a more (sub)acute inflammatory disease gathering the histological criteria to get classified as a PGI. However, despite the apparent continuum between epitheliocystis and PGI, in the study by Herrero et al. [6], PGI was only observed in farms located Southwest of Norway (as discussed above) whereas epitheliocystis was found across all 12 farms studied, suggesting that the two diseases may be due to different etiological agents or that the evolution towards typical PGI lesions may be driven by various environmental factors such as the mean water temperature or pH. In line with this 2nd hypothesis is the fact that the prevalence of epitheliocystis and the number of cysts per fish assessed histologically was associated with PGI severity and prevalence [6].

In this study, no association was observed between the presence of epitheliocystis and molecular detection of *Ca.* Piscichlamydia salmonis [6], implying the probable presence of at least one additional organism responsible for many of the observed inclusions. The microsporidian *Desmozoon lepeophtherii* was detected at increased incidence across the farms, irrespective of PGI status in fish or farms, but with higher loads in PGI-affected fish [6]. Furthermore, another study by Steinum et al. supported a multifactorial etiology for PGI, implicating *Ca.* Piscichlamydia salmonis, microsporidian, and an unidentified epitheliocystis agent as causative agents [46].

## 4. *Chlamydia*-Related Bacteria in Fish Gills

### 4.1. Gills Microbiome

The mucosal surfaces of gills and skin play important functions in fish osmoregulation, ion balance, gas exchange, and waste elimination [64]. Moreover, they are the first line of defense against microbes [65]. All mucosal surfaces of teleost fish (with mobile upper jaw) are inhabited by commensal bacterial communities, or microbiomes, which are sensitive to the environment. The dysbiosis in the gill microbiome contributes to gill pathology [66]. In addition, the mucus contains a variety of antimicrobial enzymes and peptides that protect the immune system [67,68].

### 4.2. Chlamydia-Related Bacteria as Symbionts of Fish

Both the gills and skin are major sites for host–pathogen interactions due to their large surface areas and constant contact with microbe-rich water [68]. Several studies have investigated the microbiome of the gills and shown that *Chlamydia*-related bacteria can live as symbionts of fish as they do in free-living amoebae and other eukaryotic hosts [69]. For example, *Ca.* Piscichlamydia sp. was found with a high relative-abundance in the gill-associated microbiota of the cyprinid species *Parachondrostoma toxostoma*, accounting for up to 84% of the bacteria [70]. Similarly, *Ca.* Clavichlamydia salmonicola was found to be the predominant species in the gill microbiome of Atlantic salmon during their transfer from fresh water to the sea [71]. Moreover, the microbiome diversity was found to be decreased during the transfer, which emphasizes the effects of time, water salinity, and temperature on the microorganisms colonizing the gills [71].

## 5. Permissiveness of Cell Lines Derived from Fish to Chlamydia-Related Bacteria and *Chlamydia vaughanii*

### 5.1. Waddlia chondrophila, Estrella lausannensis and Parachlamydia acanthamoebae

The potential pathogenicity of *Chlamydia*-related bacteria towards fish can be investigated by assessing the permissiveness of cell lines derived from fish for these bacteria. Kebbi-Beghdadi et al. analyzed the growth of three different *Chlamydia*-related bacteria (*Waddlia chondrophila*, *Estrella lausannensis,* and *Parachlamydia acanthamoebae*) in two fish-derived cell lines, EPC-175 (epithelial cells from fathead minnow skin) and RTG-2 (fibroblasts from rainbow trout ovary and testis) [72]. *W. chondrophila* replicated efficiently in both cell lines, with the number of DNA copies increasing by about three logs in 48 h. This bacterium also induced a strong cytopathic effect in both cell lines. *E. lausannensis* also replicated in both cell lines, but much more slowly, with only a one-log increase in the number of DNA copies in 48 h. When infected with *E. lausannensis,* no cell lysis was observed, and only a few reinfection events took place. Finally, *P. acanthamoebae* showed very limited growth in both cell lines [72].

### 5.2. Chlamydia vaughanii

*Chlamydia vaughanii* is a newly isolated chlamydia from fish and the permissiveness of different cell lines towards this bacterium was investigated in our laboratory [72]. Bacteria were able to grow efficiently at 37 °C in several mammalian cells, including human, and they were able to multiply in the fish cell line EPC-175 but only at 30 °C [73]. Conversely, insect cells (*Spodoptera frugiperda* pupa ovarian tissue cells), grown at 28 °C, and amoebae (*Acanthamoeba castellanii*), grown at 25 °C or 32 °C, did not support bacterial replication. Further investigation is needed to clarify the pathogenic potential of this novel species, its ability to create outbreaks in fish farms and its possible zoonotic capacity.

## 6. A Zebrafish Model of Infection by *Chlamydia*-Related Bacteria

### 6.1. An Attractive Model of Infection

The Zebrafish embryo attracted the attention of biologists due to its transparency almost a century ago [74]. Humans and Zebrafish share at least 70% of their coding genomes, including disease-associated genes [75]. Zebrafish embryogenesis is rapid, ex utero, and amenable to noninvasive intravital imaging and longitudinal analysis. Additionally, hundreds of embryos can be easily obtained, and pharmacological treatments can be applied by water exposure [76]. Thanks to its small size, easy breeding, genetic tractability, high fertility, and transparent larval stages, it is an attractive animal model to assess the pathogenicity of *Chlamydia*-related bacteria [77].

### 6.2. Waddlia chondrophila in Zebrafish

*W. chondrophila* is a *Chlamydia*-related bacterium that was repeatedly isolated from aquatic environments [78] and water systems [79,80,81] and is capable of infecting and replicating in fish cell lines [72]. Given this, it was hypothesized that fish could be hosts and reservoirs for *W. chondrophila* and Zebrafish were used as a model of infection by this *Chlamydia*-related bacterium [81]. Bacteria were put in contact with Zebrafish larvae by exposure in the water and they were able to cause infection in the swim bladders of the Zebrafish. Inclusions containing bacteria could be observed in the epithelium of the swim bladders and in adjacent tissues, but there was no increase in mortality by three days post-infection. However, mortality was observed when *W. chondrophila* was microinjected intravenously. Under these conditions, mortality was dose-dependent and probably due to systemic infection. Moreover, endothelial cells of the vasculature and phagocytic cells of the innate immune system were bacterial targets. The authors also observed neutrophil migration to the infection site and could demonstrate that *W. chondrophila* is taken up and can replicate inside these cells. Furthermore, this publication suggests that the myeloid differentiation factor 88 (MyD88)-mediated signaling pathway is implicated in the innate immune response to *W. chondrophila* infection.

Another Zebrafish model of infection by *W. chondrophila* revealed strong signs of infection 10 days after bacterial contact. More precisely, gills turned dark and patchy while the uninfected fish showed no pathology. Specific PCR targeting 16S rRNA gene confirmed the presence of *W. chondrophila* in the Zebrafish tail, gills, abdomen, and head [82].

## 7. Conclusions

*Chlamydia*-related bacteria are emerging as significant pathogens in aquaculture, contributing to diseases such as epitheliocystis and complex gill disease that threaten global aquaculture. Despite growing recognition of *Chlamydia*-related bacteria diversity and pathogenic potential, many aspects of their biology, host specificity, and transmission remain poorly understood, given that most of them were not successfully cultured in vitro.

Advancements in molecular diagnostics, genomics, and experimental models like Zebrafish could offer promising roads to unravel *Chlamydia*-related bacteria–host interactions and figure out effective treatment strategies. The availability of a new species within the *Chlamydia* genus (*C. vaughanii*), that naturally infects fish, represents a unique opportunity to establish a relevant Zebrafish model of infection with a *Chlamydia*. Not only can the infection of Zebrafish with *C. vaughanii* be used to test various Zebrafish mutants and uncover host genes important in chlamydial pathogenesis in fish, but this model might also benefit from recent advances in the genetic transformation of members of the *Chlamydia* genus.

Continued research will help to precise the prevalence of aquatic chlamydiae in different water environments as well as their impact on fish health and on health of other water inhabitants such as free-living amoebae, flagellates, ciliates, and molluscs. Continued research is also essential to ensure the sustainability of aquaculture.

## Data Availability

No new data were created or analyzed in this study. Data sharing is not applicable to this article.
